# Supplementary material for: Time-dependent computational model of post-traumatic osteoarthritis to estimate how mechanoinflammatory mechanisms impact cartilage aggrecan content
Source: PLoS Comput Biol. 2025 Oct 31;21(10):e1013641. doi: 10.1371/journal.pcbi.1013641 (PMC12594405; doi:10.1371/journal.pcbi.1013641)
Supplement: Attachment — Submitted filename: Response to Reviewers.docx [file pcbi.1013641.s003.docx]

**Manuscript number: PCOMPBIOL-D-24-01918, revision number: 1**

*Time-dependent computational model of post-traumatic osteoarthritis to estimate
how mechanoinflammatory mechanisms impact cartilage aggrecan content*

**RESPONSES TO THE REVIEWERS´ COMMENTS**

The Authors express their deepest gratitude to the Reviewers for the considerable time and effort taken in the review of our manuscript. We appreciate your highly encouraging feedback, constructive comments, and recommendations for the improvement of the document. The parts that we have changed in the revised manuscript with track changes are marked with a yellow background.

**REVIEWER #1**

**GENERAL COMMENT:** In this manuscript, the authors develop a coupled mechanical and biological model that predicts aggrecan depletion in explants with and without an impact-induced focal lesion under cyclic loading. The mechanical model is well-established, using the porohyperviscoelastic framework and material constants previously employed by the research group for explants and whole joints. The biological model includes cell damage that induces aggrecanase release (causing proteolytic decreases in aggrecan content), fluid-flow driven aggrecan release, and pressure-driven aggrecan synthesis. Model parameters are obtained from a combination of prior literature and model fits; a sensitivity study was used to determine that the initial aggrecan content and lesion geometry are the most critical parameters to have accurate values for. The overall model results recapitulate experimental results, showing greater aggrecan depletion near lesions.

Overall, the manuscript and the approach have many strengths. The approach, while having parameters that are not well-established in the literature, is thoroughly documented with references and support provided to the extent available. The model is rigorously developed, including mesh sensitivity studies, parameter studies, and evaluation of sensitivity to geometry. Despite these strengths, there are a few overarching concerns that should be more completely addressed in the manuscript.

**AUTHOR RESPONSE:** Thank you for the positive feedback on our work. Responses to the concerns as well as the corresponding author actions are provided below.

**REVIEWER COMMENT (1):** First, the aggrecan focus is narrow; the authors have prior work that also focuses on collagen failure. Why is collagen failure not included in this model?

**AUTHOR RESPONSE:** Our focus on aggrecan was motivated by this manuscript’s emphasis on the earliest mechanoinflammation-associated events in the progression of PTOA. Collagen failure has often been implemented as a *purely mechanical* process over model iterations of *arbitrary* time [1,2]. To complement previous work, we specifically focused here on the development of a *cell-driven* *mechanoinflammatory*, real/continuous *time-dependent* model (12 days) compared against experimentally observed cartilage composition. We started this development with aggrecan-only focus due to enzymatic aggrecan loss reported to precede enzymatic degradation and loss of collagen in the early time points [3,4] and due to readily available spatio-temporal aggrecan data [5]. We fully acknowledge that collagen failure (as well as fatigue [2] and enzymatic degradation [6]) are critically important in the longer-time kinetics and progression of OA/PTOA; the development of corresponding mechanoinflammatory model and collection of collagen content data are our ongoing activities.

In more detail, injurious overloading may result in collagen failure (*e.g.*, excessive strain in fibril direction) [1,7,8]. Collagen failure and matrix softening may be implemented by updating material properties at certain time points, the selection of which may be challenging to justify. Rigorous selection of the time parameters would necessitate biomechanical tests at different time points, obtaining collagen content maps, and estimating parameters for matrix metalloproteinase activity, collagen denaturation, and aggrecan–collagen interaction [4,8]. Hypothetically, collagen failure in such a framework would result in locally higher strains, more cell damage, and more aggrecan loss; thus, the currently presented parameters are an estimate for the upper limit of mechanoinflammatory aggrecan degradation in a 12-day setup. Including collagen becomes increasingly important in joint-level models simulating months/years of progression [2].

**AUTHOR ACTION:** We added more discussion on collagen failure and motivation behind the study.

*Introduction* – *(page 5, lines 101–102):* “These models also implemented the decrease of cartilage load-bearing capability by iteratively updating permeability, matrix stiffness...”

*Introduction* – *(page 6, lines 114–116):* “Specifically, the framework utilizes time-dependent partial differential equations instead of an iterative approach with arbitrary time between model iterations.”

*Discussion* – *(pages 17–18, lines 383–393):* “Degradation softens cartilage, potentially leading to locally increasing strains over time. This has been captured in previous models focusing on collagen failure, aggrecan depletion, and permeability increase [7,50] by using an iterative approach, where ABAQUS material model was updated in each iteration (arbitrary unit of time, necessitating the introduction of new parameters). Rigorous implementation of collagen failure to the current time-dependent mechanoinflammatory model would necessitate use of biomechanical tests, collagen content maps, and estimates of matrix metalloproteinase activity at several time points which is a topic for future work. (…) While tissue softening may not be a significant factor over 12 days, it becomes important over longer times in joint-level models.”

**REVIEWER COMMENT (2):** Second, the focus on immature cartilage samples hampers translational ability. While I appreciate that mature tissue can be more challenging to obtain (and more heterogeneous to work with), further discussion on what the steps are to translate this work into predicting aggrecan depletion in human cases would be useful (i.e., line 248-249 is insufficient).

**AUTHOR RESPONSE:** Thank you for bringing up the important translational aspect. Indeed, immature bovine provided here a homogeneous sample source to build the experimental model. It would be almost impossible to get a homogeneous set of adult human samples, especially relatively healthy samples when investigating early changes following injury.

To translate aggrecan depletion estimations/simulations to (mature) humans, the first step is to explore mechanoinflammation with new explant culture studies using mature human (cadaver) cartilage and organ-on-a-chip models with human cells. These data could be used to calibrate the cell–tissue-level numerical models. Next, the modeling framework should be expanded to the knee joint-level, where simulated aggrecan loss could be compared for example against clinical data comprising of quantitative MRI and studies of matrix biomarkers in synovial fluid after injury.

**AUTHOR ACTION:** Steps to translate these findings to human *in vivo* were added.

*Discussion* – *(page 17, lines 367–382):* “… it must be acknowledged that the present numerical estimates of mechanoinflammatory processes — with some model parameters based on prior computational models when experimental evidence was unavailable — may not be fully translatable to more mature large animal or human cartilage. (…) Therefore, new explant culture/bioreactor studies should be conducted, not only with immature cartilage but also with mature (human cadaver) cartilage, in order to fully calibrate the numerical representation of the underlying mechanoinflammation. Another approach is organ-on-a-chip models with human cells, providing added control over desired mechanobiological processes compared to *ex vivo* models [80,81]. To further promote the translational ability of the model to the knee joint-level, the simulated aggrecan depletion should be confirmed in joint-level mechanoinflammatory models (*e.g.*, comparison to quantitative MRI maps at baseline and several follow-up time points [59]) and compared against matrix biomarkers released to synovial fluid or systemic circulation [35,82]. Ultimately, such models could be utilized to design various rehabilitation exercises or tissue-engineered constructs to tackle disease progression.”

**REVIEWER COMMENT (3):** Third, the lack of experimentally-based constants is a real limitation of the literature; and the fact that some of the references are prior modeling studies (not experimental studies) should be made clear in the text.

**AUTHOR RESPONSE & ACTION:** We fully agree with the Reviewer. We clarified the text regarding the use of references to computational studies for some of the model parameters.

*Methods* – *(page 26, lines 568–572):* “Due to limited experimental evidence for the model parameter values, a sensitivity analysis was conducted (…). The parameter value ranges were selected based on computational and experimental studies …”

*Discussion* – *(page 13, lines 266–269):* “The strain threshold $\varepsilon_{dmg,init}=$ 40% for initiating damage was based on computational studies [47,59] but it is noteworthy that also lower shear strains of 7–12%, as observed under shear loading of immature cartilage, have been associated with cell damage/death [57,67].”

*Discussion* – *(page 14, lines 304–306):* “The selected fluid flow velocity threshold with reasonable model fit (0.08 $mm\cdot s^{-1}$) was higher than a previous computationally estimated [7] value of 0.04 $mm\cdot s^{-1}$.”

*Discussion* – *(page 15, lines 317–319):* “Increasing the basal aggrecan biosynthesis rate only in healthy cells by up to 100% (from experiments: 66–95% [71], 20–120% [40]; ^35^S-sulfate incorporation assays after similar CL protocols)…”

*Discussion* – *(page 17, lines 367–370):* “… it must be acknowledged that the present numerical estimates of mechanoinflammatory processes — with some model parameters based on prior computational models when experimental evidence was unavailable — may not be fully translatable to more mature large animal or human cartilage.”

*Methods* – *(page 23, lines 511–512):* “… where thresholds for cell damage initiation $\varepsilon_{dmg,init}=40\%$ [47,55,59] and for maximum damage $\varepsilon_{dmg,max}=150\%$ [55] (obtained from computational studies).”

*Methods* – *(page 25, lines 559–564):* “… in areas with healthy cells [9] experiencing moderate fluid pressure alterations. Here, the rate of change of fluid pressure considered to be in the moderate/healthy range of 20–60 $MPa\cdot s^{-1}$ (ramp-up time 0.2s of the cyclic loading, 1 Hz, 40% duty cycle) corresponds to intermittent peak pressures in the range of 4–12 MPa that have been linked to anabolic responses in experiments [22,58,63–65]).”

**REVIEWER COMMENTS (4–5):** The second paragraph is particularly well-written. The introduction overall is excellent. Line 102 “are presently few but” appears to be a typo.

**AUTHOR RESPONSE & ACTION:** The Authors thank the Reviewer for the praises. The typo has been fixed.

*Introduction* – *(page 5, lines 103–106):* “While the number of models incorporating cell-level responses (...) is currently limited, they provide important advancements in numerical estimation of mechanoinflammation.”

**REVIEWER COMMENT (6):** Section 2.2: a pointer to the table with constants would be helpful here, as thresholds are mentioned but not given (*e.g.*, “excessive shear strain: in line 136)

**AUTHOR RESPONSE & ACTION:** Certainly. Pointers to the corresponding Methods section and table have been added.

*Results* – *(page 7, lines 141–143):* “… in regions of excessive shear strain at the time of maximum compression, ‘healthy’ cells were assumed to switch their phenotype to ‘damaged’ cell (only these two cell populations were implemented; see Section 4.6, Table 1, and Supplementary Table S2).”

**REVIEWER COMMENT (7):** Some of the threshold references do not seem applicable. For example, reference 61 does not provide a fluid pressure that would cause a response, it instead evaluates when behavior is incompressible.

**AUTHOR RESPONSE & ACTION:** The Reviewer is correct, this was an oversight from us. The reference list has been double-checked and updated for more relevant literature. Instead of the previous reference [61]

- Bachrach *et al.* (1998), *J Biomech*, “Incompressibility of the solid matrix of articular cartilage under high hydrostatic pressures”,

the updated manuscript and supplementary material text and tables now refer to

- Torzilli *et al.* (1999), *J Biomech Eng*, ”Effect of impact load on articular cartilage: Cell metabolism and viability, and matrix water content”,
- Milentijevic & Torzilli (2005), *J Biomech*, “Influence of stress rate on water loss, matrix deformation and chondrocyte viability in impacted articular cartilage”,
- Elder & Athanasiou (2008), *PLoS One*, “Synergistic and additive effects of hydrostatic pressure and growth factors on tissue formation”
- Parkkinen *et al*. (1993), *Arch Biochem Biophys*, “Effects of Cyclic Hydrostatic Pressure on Proteoglycan Synthesis in Cultured Chondrocytes and Articular Cartilage Explants”.

*Results* – *(pages 8–9, lines 173–177):* “… injury-associated cell damage and subsequent catabolic cell activity (aggrecanase release) were investigated [3,22,45,55–58]: (…), rapid change of fluid pressure over the time of injurious compression (threshold: $80 MPa\cdot s^{-1}$ which over 0.5 s of ramping up the INJ loading corresponds to peak pressure in the range of ~40 MPa [22,58,60,61], Fig. 3C)…”

*Results* – *(page 10, lines 208–211):* “… acceleration of aggrecan biosynthesis by up to 100% from basal biosynthesis rate in regions where fluid pressure changes were within a healthy range (20–60 $MPa\cdot s^{-1}$ which over 0.2s of ramping up the CL (1 Hz, 40% duty cycle) corresponds to peak pressures in the range of 4–12 MPa [22,58,63–65]; Fig. 5C).”

*Methods* – *(page 25, lines 559–564):* “… $R_{\mathrm{biosynthesis}}$ depended on the change of fluid pressure over time in areas of healthy cells [9] experiencing moderate fluid pressure alterations. Here, the rate of change of fluid pressure considered to be in the moderate/healthy range of 20–60 $MPa\cdot s^{-1}$ (ramp-up time 0.2s of the cyclic loading, 1 Hz, 40% duty cycle) corresponds to intermittent peak pressures in the range of 4–12 MPa that have been linked to anabolic responses in experiments [22,58,63–65]).”

**REVIEWER COMMENT (8):** Lines 258-262: why is the strain damage threshold larger in the model (40%) than in prior studies (7-12%)? Is this an artifact of other errors in the model?

**AUTHOR RESPONSE:** The 40% threshold (logarithmic strain obtained from previous *computational* studies), loading protocol as in the experiment, and the used material model together resulted in comparable aggrecan loss responses as in literature. Thus, the higher threshold (40% *vs.* 7–12% [9]) to produce these results is not an artifact of model errors *per se*, but a *consequence of the modeling assumptions* (including potential differences in depth-wise collagen content and orientation in the immature tissue modeled here in 2D *vs.* in the experiments of Ayala *et al*. [9]). In contrast, the 7–12% shear strain was from an *experimental* study (shear loading conditions under confocal elastography, engineering strain), providing a discussion topic that already substantially lower levels of shear strain have been *suggested to* *initiate* cell damage/death. To better accommodate the shear strain–cell damage responses, a plausible approach could be to implement probability density functions to 3D modeling frameworks. In the current model, decreasing the threshold would manifest as increased aggrecanase production (already included in Fig. 4C).

**AUTHOR ACTION:** Discussion about the shear strains modified.

*Discussion* – *(page 13, lines 266–273):* “The strain threshold $\varepsilon_{dmg,init}=$ 40% for initiating damage was based on computational studies [47,59] but it is noteworthy that also lower shear strains of 7–12%, as observed under shear loading of immature bovine cartilage, have been associated with cell damage/death [57,67]. This discrepancy originates from the modeling assumptions (*e.g.*, compressive loading conditions, material model including collagen orientation implemented in 2D) *vs.* the real tissue in the shear experiments [57,67]. To better accommodate the different shear strain–cell damage responses, a more probabilistic approach with probability density functions could be undertaken in the future. Here, using a lower strain threshold value…”

**REVIEWER COMMENT (9):** Lines 274-275: why use axial strain (which depends on coordinate system) rather than a principal strain (which is not dependent on coordinate system)?

**AUTHOR RESPONSE:** The Reviewer is correct, the (minimum) principal strain would be a more generalized output across different model geometries than (logarithmic) compressive axial strain. In the current study with simple compressive loading of 2D geometries, both strain measures provide similar strain estimates (peak strain 2%-points higher in minimum principal *vs.* axial strain model, see Fig. on the right), thus, simulated aggrecan losses are the same. We modified the text to consider that in simple geometries, both axial and minimum principal strain provide a reasonable basis to trigger cell damage in this model, but in more complex geometries the coordinate system-independent strain measures would be the go-to solution (principal and maximum shear strains).

**AUTHOR ACTION:** Text modified to consider also (minimum) principal strains instead of axial strain-only.

*Discussion* – *(page 14, lines 289–294):* “… that coordinate system-dependent logarithmic axial strain (Fig. 3D; comparable to minimum principal strain in simple geometries, Supplementary Fig. S2) and fluid pressure change over time (Fig. 3C) could be used to trigger cell damage and replicate the experimental findings. The axial strain is usable in simple geometries and loading conditions, while the shear strain, minimum principal strain, and fluid pressure could serve as viable computational biomarkers of cell damage in more complex joint-level models [70].”

*Supplementary Material S1* – Added the above logarithmic axial *vs.* logarithmic minimum principal strain figure

**REVIEWER COMMENT (10):** Line 366: is there ever lift-off in the bioreactor?

**AUTHOR RESPONSE & ACTION:** Yes there was for the rest periods. Regarding potential lift-off during the active part of cyclic loading protocol, our choice of using haversine instead of sine waveform was motivated by physiological relevance. The result of using haversine waveform was minimization of lift-off. However, we cannot rule out the possibility of near-lift-off situations or distorted waveforms for some of the samples, as reported previously [10–12].

*Methods* – *(pages 19–20, lines 426–435):* “After establishing repeatable contact with the explants with 10% compressive offset strain (...), the explants were subjected to cyclic unconfined compression with 15% axial strain (1 Hz, haversine waveform, 40% duty cycle, 1-h loading sessions followed by 5-h rest with the applied load removed; this protocol was repeated four times a day) in an incubator-housed cyclic loading apparatus [7,13,14]. Our choice to use haversine instead of sine waveform was motivated by physiological relevance, and the use of haversine waveform resulted in minimization of lift-off of the contact surface from cartilage surface during the active part of cyclic loading [39,40,87]. However, we cannot rule out the possibility of near-lift-off situations for some of the samples.”

**REVIEWER COMMENT (11):** Section 4.5: a recent publication described the potential errors in FE-predicted stress and strain in cartilage explants as a result of imperfections in the samples (<https://doi.org/10.1016/j.jbiomech.2024.112323>). Were your models idealized, and how does that compare to their actual geometry?

**AUTHOR RESPONSE:** Great point by the Reviewer, our models were indeed idealized. The model assumed perfectly flat and horizontal surface and 1-mm-thick geometry. In comparison, the actual geometries may have surface roughness, the top surface angle may slightly vary from sample-to-sample (compared to the bottom surface angle), and even though the cartilage explants were trimmed to 1 mm, they could have become thicker by up to 50 μm (5%) [13,14] due to swelling during culture. Furthermore, the shapes of the modeled lesions, as obtained from histology, were implemented on idealized geometry with horizontal surface (see also our response to the comment (12) by the Reviewer). The model idealizations may decrease the heterogeneity in the stress/strain fields [15], thus resulting in relatively symmetrical aggrecan content losses on both sides of the lesion.

**AUTHOR ACTION:** Methods section and the limitations paragraph in the Discussion were updated.

*Methods* – *(page 22, lines 481–483):* “… the sizes and shapes of the lesions were sketched with the spline tool on intact geometry (3-mm-wide by 1-mm-thick rectangle). These idealized intact and injured geometries were meshed in ABAQUS.”

*Discussion* – *(page 16, lines 353–358):* “Another limitation is the use of an idealized model geometry with perfectly flat and horizontal cartilage surface, and constant thickness, as opposed to experimental samples that may have had uneven surfaces and non-constant thicknesses (Fig. 1B). This idealized model geometry has been suggested to reduce heterogeneity in strain fields during loading (and, thus, in simulated adaptation responses, Fig. 7) when compared to models with sample-specific geometries [77].”

*Discussion* – *(page 17, lines 371–373):* “… use of different loading amplitudes and frequencies, sample-specific geometries, and larger sample sizes could improve the robustness of the model parameters across different loading scenarios.”

**REVIEWER COMMENT (12):** Section 4.5: how was the lesion modeled? (And, in section 4.6, how were the five lesion models generated?)

**AUTHOR RESPONSE:** The model presumes an initial lesion caused by the injurious loading. When these models with histology-based lesion geometries were subsequently subjected to compressive loading (“injury + cyclic loading model”), the lesion size and shape were unaltered. Such lesion formation or propagation modeling frameworks were omitted in this study (please also see our response to comment (3) by Reviewer #3). The lesion sizes and shapes were segmented from the histological sections of five different cartilage plugs included in the study.

**AUTHOR ACTION:** Methods section was updated.

*Methods* – Section 4.5 – *(pages 21–22, lines 478–485):* “Lesion formation or crack propagation [78] were not modeled. Instead, the conventional light microscopy images of the cartilage sections were imported to 3D Slicer (v. 5.4.0) [90] for manual segmentation of five representative lesion geometries of varying size and shape (five different plugs). The geometries were then imported to ABAQUS, where the sizes and shapes of the lesions were sketched with the spline tool on intact geometry (3-mm-wide by 1-mm-thick rectangle). These idealized intact and injured geometries were meshed in ABAQUS. Cyclic loading was subjected to intact (CL group) or injured geometry (INJ+CL group, five lesion geometries based on the cartilage sections, Fig. 6D).”

*Methods* – Section 4.6 – *(page* 26*, lines 574–578):* “… and five different lesion geometries were investigated. These lesion models were generated by (1) segmenting five lesion geometries from images of histological sections cut from five different injured cartilage plugs, then (2) subtracting the segmented lesion geometry from the superficial–middle region of an intact rectangle (see Section 4.5).”

**REVIEWER COMMENT (13):** Figure 1: how far away from the center of the lesion is “away from lesions”?

**AUTHOR RESPONSE & ACTION:** Distance of “away from lesion” region from the center of the lesion was added, and the whole sentence was clarified.

*Methods* – *(page 20, lines 446–452):* “… optical density was measured in the following regions: … away from lesions (… mean distances (±95%CI) from lesion edges to away-from-lesion region edges 653±97 μm, and from lesion center-points to away-from-lesion region edges 743±101 μm).”

**REVIEWER #2**

**REVIEWER COMMENT (1):** This was an interesting, comprehensive manuscript. My main concern relates to the mechanoinflammatory model: This is a novel aspect of the work and I found section 4.6 lacking in detail for me to appreciate this modelling component. I think the supplementary material S3 would be better placed in the main body of the manuscript. [S1 &S2 fine in the supplementary material]

**AUTHOR RESPONSE:** Thank you for the positive feedback. We decided to add more details about the novel parts of the mechanoinflammatory model to the main manuscript, but wish to leave *the full model description* with 9 equations, 2 pages of text, and 2.5-page-long parameter table to the Supplementary Material, as inflating the main manuscript with technical details would make the work more complicated for a general reader. In turn, we added *a new summarizing figure* to accompany the *text additions* (also as a response to the comment (2) by the Reviewer), as well as *a new summarizing table* about the main cell damage and aggrecan loss terms, and related parameters in the different models. We hope our choice is satisfactory for the Reviewer.

**AUTHOR ACTION:** Parts of the Supplementary Material S3 (“Mechanoinflammatory model”) have been added to the main manuscript (regarding cell damage, aggrecanase release, aggrecan depletion due to excessive fluid velocity and aggrecan biosynthesis). All references to equations have been updated. A new, summarizing figure (Fig. 7) and new table (Table 1) have been added.

*Opening line of Discussion* – *(page 12, lines 237–239):* “We developed time-dependent computational models of cartilage mechanoinflammation to estimate changes in spatial aggrecan content under injurious and physiological cyclic loading (summarized in Fig. 7).”

*Methods* – *(pages 24–25, lines 535–567):* “The damaged cell population $C_{cell,damaged}$ evolved over time as

$\frac{\partial C_{cell,damaged}}{\partial t}=k_{\mathrm{cl}} f_{\mathrm{dmg}}(\varepsilon)C_{cell,healthy}$,

(4)

where $k_{\mathrm{cl}}$ is the rate of cells turning from healthy to damaged due to cyclic loading and$C_{cell,healthy}$ is the concentration of healthy cells. The presence of damaged cells gave rise to localized, time-dependent catabolic stimulus for aggrecanase release $S_{\mathrm{aga}}$. The introduction of a stimulus variable — that has time-dependent rate coefficients to capture the time delay in mechanoinflammatory responses but no diffusion *per se* — was motivated by Kar *et al.* [62] and experimental findings [4,69]. The latter includes observations that cell-driven enzymatic activity is delayed (in contrast to very early aggrecan loss due to structural microdamage from the mechanical insult) and increases after hours–days from injury [69] (delay due to trauma-related release of alarmins and damage-associated molecular patterns peaking in ~24h [4]).

$\frac{\partial S_{\mathrm{aga}}}{\partial t}=\alpha_{\mathrm{aga}}\left( k_{\mathrm{aga}}C_{cell,damaged}- S_{\mathrm{aga}} \right)$,

(5)

where $\alpha_{\mathrm{aga}}$ is rate constant for aggrecanase stimulus, and $k_{\mathrm{aga}}$ is constant for aggrecanase release from damaged cells. The catabolic stimulus $S_{\mathrm{aga}}$ was linked to the production of aggrecanases (that diffuse through the extracellular matrix) as

$\frac{\partial C_{\mathrm{aga}}}{\partial t}={D_{\mathrm{aga}}^{*}e^{-d_{1}C_{\mathrm{agg}}}\nabla}^{2}C_{\mathrm{aga}}+k_{1}S_{\mathrm{aga}}-k_{2}C_{\mathrm{aga}}$,

(6)

where $D_{\mathrm{aga}}^{*}$ is diffusivity of aggrecanase, $d_{1}$ is a constant defining the dependency of aggrecanases’ effective diffusivity on local aggrecan concentration ($D_{\mathrm{aga}}=D_{\mathrm{aga}}^{*}e^{-d_{1}C_{\mathrm{agg}}}$), $k_{1}$ is rate constant for generating aggrecanases, and $k_{2}$ is enzymatic loss of aggrecanases. Finally, the aggrecan concentration $C_{\mathrm{agg}}$ evolved as:

$\frac{\partial C_{\mathrm{agg}}}{\partial t}={D_{\mathrm{agg}}\nabla}^{2}C_{\mathrm{agg}}-R_{\mathrm{proteolytic}}-R_{fluid flow}+R_{\mathrm{biosynthesis}}$,

(7)

where (…). The sink/source terms $R_{fluid flow}$ and $R_{\mathrm{biosynthesis}}$ (Table 1) included the main loading-related variables (fluid velocity and change of fluid pressure over time, respectively) as normalized, threshold-based functions (same form as Eq. (1), see Supplementary Material S1, Section S3).”

*Methods* – *[new]* *Table 1:*

**Table 1. Summary of the mechanoinflammatory equations and parameters in the four different models.** For the full model description of the cell damage and aggrecan loss, see Section 4.6 and Supplementary Material S1, Section S3. CTRL = free-swelling control, INJ = injurious loading, CL = cyclic loading. Variable $z$ is normalized depth in cartilage ($z=0$ surface, $z=1$ bottom) and $H=1$ mm is cartilage thickness.

| **Variable / Parameter** | **Expression / Value** | **Description / Ref.** |
| --- | --- | --- |
| **Damaged cells**  $\boldsymbol{C}_{\mathbf{cell}\mathbf{,}\mathbf{damaged}}$ **[**$\mathbf{cells}\boldsymbol{\cdot}\mathbf{m}^{\boldsymbol{-}\boldsymbol{3}}$**]** |  |  |
| In CTRL model | $C_{cell,damaged}=0$ | No damaged cells [62] |
| In INJ model | $C_{cell,damaged}= C_{cell,damaged,init}=k_{\mathrm{inj}}f_{\mathrm{dmg}}(\varepsilon)C_{cell,healthy,init}$ | Eq. (2) [53,75]. On day 0, healthy cells turn damaged as per damage function $f_{\mathrm{dmg}}(\varepsilon)$ in Eq. (1), with shear strain thresholds $\varepsilon_{dmg,init}=40\%$ and $\varepsilon_{dmg,max}=150\%$[47,55,59]. Cell damage is linked to proteolytic activity via Eqs. (5) and (6). |
| In CL model | $\frac{\partial C_{cell,damaged}}{\partial t}=k_{\mathrm{cl}} f_{\mathrm{dmg}}(\varepsilon)C_{cell,healthy}$, with  $C_{cell,damaged,init}=0$ | Eqs. (4) and (S14). Rate of strain-driven cell damage during cyclic loading, without initial cell damage |
| In INJ+CL model | $\frac{\partial C_{cell,damaged}}{\partial t}=k_{\mathrm{cl}} f_{\mathrm{dmg}}(\varepsilon)C_{cell,healthy}$, with  $C_{cell,damaged,init}=k_{\mathrm{inj}}f_{\mathrm{dmg}}(\varepsilon)C_{cell,healthy,init}$ | Eqs. (4) and (S14). Rate of strain-driven cell damage during cyclic loading, with initial cell damage from injurious loading of day 0 |
| **Aggrecan loss reaction terms**  **in Eq. (7) [**$\boldsymbol{mol\cdot}\mathbf{s}^{\boldsymbol{-1}}\boldsymbol{\cdot}\mathbf{m}^{\boldsymbol{-3}}$**]** |  |  |
| $R_{\mathrm{proteolytic}}$ in CTRL | 0 | No proteolytic activity |
| $R_{\mathrm{proteolytic}}$ in CL, INJ, INJ+CL | $k_{3}C_{a\mathrm{ga}}\frac{C_{a\mathrm{gg}}}{C_{a\mathrm{ga}}+K_{m,aga}}$ | Eq. (S17) [62]. Rate of change of aggrecan concentration $C_{\mathrm{agg}}$ due to aggrecanases $C_{\mathrm{aga}}$ |
| $R_{fluid flow}$ in CTRL, INJ | 0 | No fluid flow-driven aggrecan depletion in the absence cyclic loading over 12 days |
| $R_{fluid flow}$ in CL, INJ+CL | $k_{cl,fl} f_{\mathrm{dmg}}(v)C_{a\mathrm{gg}}$ | Eq. (S18). Rate of change of aggrecan concentration $C_{\mathrm{agg}}$ due to elevated fluid flow as per damage function $f_{\mathrm{dmg}}(v)$, with similar form as Eq. (1), and fluid velocity thresholds $v_{dmg,init}=0.08 mm\cdot s^{-1}$and $v_{dmg,max}=0.15 mm\cdot s^{-1}$ [7,47]. |
| $R_{\mathrm{biosynthesis}}$ in CTRL, INJ | $P_{\mathrm{ag}}\left( 1+0.9\frac{1-z}{H} \right)C_{cell,healthy}\left( 1-\frac{C_{a\mathrm{gg}}}{C_{agg,tar}} \right)$ | Eq. (S20). Rate of change of aggrecan concentration $C_{\mathrm{agg}}$ due to aggrecan biosynthesis, with homeostatic target aggrecan concentration $C_{agg,tar}$[7,62]. |
| $R_{\mathrm{biosynthesis}}$ in CL, INJ+CL | $\left( 1+f_{s\mathrm{ynth}}\left( \hat{p} \right) \right)P_{\mathrm{ag}}$  $\cdot\left( 1+0.9\frac{1-z}{H} \right)C_{cell,healthy}\left( 1-\frac{C_{a\mathrm{gg}}}{C_{agg,tar}} \right)$ | Eq. (S20). Rate of change of aggrecan concentration $C_{\mathrm{agg}}$ due to aggrecan biosynthesis accelerated due to CL as per normalized biosynthesis function $f_{\mathrm{synth}}\left( \hat{p} \right)$, with similar form as Eq. (1), and fluid pressure change thresholds $\hat{p}_{synth,init}=20 MPa\cdot s^{-1}$ and $\hat{p}_{synth,max}=60 MPa\cdot s^{-1}$ [22,58,63–65]. |
| **Parameters** |  |  |
| $k_{\mathrm{inj}}$ [-] | 0.45 | Eq. (2) [16,53]. Fraction of damaged cells after injury |
| $k_{\mathrm{cl}}$ [${10}^{-6}$ $s^{-1}$] | 1.5 | Eqs. (4), (S13), and (S14), model fit. Rate of strain-driven cell damage during cyclic loading |
| $k_{3}$ [$s^{-1}$] | 0.9 | Eq. (S17) [62]. Catalytic rate constant for aggrecanase to degrade aggrecan |
| $k_{cl,fl}$ [${10}^{-6}$ $s^{-1}$] | 1.5 | Eq. (S18), model fit. Rate of fluid flow-driven aggrecan depletion |
| $K_{m,aga}$ [$\mathrm{mol}\cdot m^{-3}$] | $5.5\cdot{10}^{-5}$ | Eq. (S17) [62,93,94]. Michaelis constant for aggrecanase |
| $P_{\mathrm{ag}}$ [$mol\cdot\mathrm{cell}^{-1}\cdot s^{-1}$] | $2.4\cdot{10}^{-22}$ | Eq. (S20) [62,95,96]. Basal aggrecan biosynthesis rate (from healthy cells) |

**REVIEWER COMMENT (2):** The results section included temporal evolution of the aggrecan content, however didn't include temporal evolution of other quantities in the mechanoinflammatory (MI) model. It would be helpful to present temporal evolution of all variables in the MI (perhaps as supplementary material if it doesn't seem appropriate to put in main body of text)

**AUTHOR RESPONSE:** Thank you for the excellent suggestion, we added a new summary figure showcasing the temporal changes in the main variables (Fig. 7, below). This new figure is organized the same way as the model is described in the updated manuscript (after parts of the Supplementary Material were moved to the main manuscript as suggested in the previous Reviewer comment): cell damage, released aggrecanases, and aggrecan content (catabolic stimulus map is omitted since it looks visually very similar to cell damage map; as per Eq. (5), $S_{\mathrm{aga}}$ depends on $C_{cell,damaged}$ and it has no diffusion coefficient). The new figure complements the original manuscript, where the terms describing fluid velocity and biosynthesis effects were already shown (Fig. 5E).

**AUTHOR ACTION:**

*Discussion* – *(page 14, lines 297–303):* “Simulated 12-day CL on injured cartilage resulted in progressive and localized cell damage [64] but, as expected, without noticeable bulk damage in addition to the INJ only (Figs. 3A, 5E, 7A). The associated bulk proteolytic activity from INJ only increased rapidly over the first three days (Fig. 7B), but with CL the released aggrecanase concentration increased only in very localized near-lesion areas compared to away-from-lesion (magenta highlight in Fig. 7B). Thus, the proteolytic activity alone did not capture the observed aggrecan loss in the INJ+CL model (Fig. 5D, right panel), necessitating the addition of the fluid flow mechanism…”

*Discussion* – *(pages 14–15, lines 308–314):* “…fluid flow dominates over proteolytic activity in the depletion of aggrecan fragments near lesions during CL… Modeling-wise this relatively low near-lesion proteolytic activity stems from using the same shear strain threshold in both INJ and CL (small near-lesion area with additional damaged cells) and setting aggrecanase concentration to zero on boundaries [62]. The latter results in high concentration gradient for aggrecanases (fast diffusion) and, thus, low aggrecanase content around the lesion (Fig. 7B).”

*Discussion* – *(page 15, lines 317–321):* “Increasing the basal aggrecan biosynthesis rate only in healthy cells by up to 100% (…) partially counteracted the depth-wise proteolytic activity in deep and intact regions (Fig. 5E, right panel) and resulted in higher bulk aggrecan content in the INJ+CL model compared to the INJ model on day 12 (Fig. 7C).”

**Fig. 7 [new figure]. Summary of the time-dependent mechanoinflammatory responses after injurious overloading. (A)** Cell damage was observed throughout the cartilage. Physiological cyclic loading (red dashed line) increased the fraction of damaged cells over time near the chondral lesion compared to injury-only. The cell damage maps as shown here were similar for the catabolic stimulus variable as per Eq. (5). **(B)** The aggrecanase content (released from damaged cells) increased rapidly in the first three days after injury. Cyclic loading further increased aggrecanase content but only in very localized areas near the lesion compared to away-from-lesion (magenta color; “localized near” region is from the superficial near-lesion region showing high cell damage, “localized away” is the same region translated to away from lesion). **(C)** The aggrecan content was lower near the lesion compared to away-from-lesion. The rate of aggrecan loss near the lesion increased when cyclic loading was involved, especially in the first three days. By day 12, the average aggrecan content in the bulk tissue was higher in the injury and cyclic loading model compared to injury-only model, highlighting the overall beneficial effect of cyclic loading. Note that the aggrecan content was regulated not only by aggrecanases, but also by fluid velocity and aggrecan biosynthesis (see Fig. 5E).

**REVIEWER COMMENT (3):** A minor point on the MI model - there is no consideration of basal production/loss terms for the healthy/damaged cell populations. Over the time-scales of application of the model this doesn't seem relevant, however some comment here would be helpful why such terms are omitted - and if those terms may be relevant for modelling OA progression or rehabilitation modelling over longer timescales.

**AUTHOR RESPONSE:** We omitted the *basal* sink/source terms of the cell populations (rate parameters set to zero as done in Kar *et al.* [6]) due to their potential irrelevance in comparison to *overloading-related* terms (*e.g.*, right-hand side of Eq. (4)) especially over short time scales. This choice stem from our study design where we were more interested in between-group responses to explore the disease mechanisms (control *vs.* injury *vs.* injury + cyclic loading). Moreover, the model provided reasonable aggrecan content estimates even without the added complexity of defining new rate parameters. We agree with the Reviewer that over longer time scales the basal rate parameters become more relevant (as well as defining more cell populations, see Comment (1) by Reviewer #3) for OA progression/rehabilitation/anti-oxidative drug modeling, which is a part of our ongoing work.

**AUTHOR ACTION:** Added text to the Discussion.

*Discussion* – *(pages 15–16, lines 332–347):* “The affected cell populations may orchestrate time-dependent reparative/remodeling responses to injury such as upregulating production of ADAMTS and tissue inhibitors of matrix metalloproteinases in surrounding viable cells (healthy and/or damaged) and, depending on the microenvironment, switching damaged cells to dead or back to healthy phenotype [4]. Modeling-wise the cellular variables and associated production/loss rates for different cell populations (as in Eq. (4)) may be implemented as linked to a certain, hypothesized mechanism or as basal sink/source terms when the real mechanism is not fully known. (…) While the basal cell production/loss terms might become more relevant in longer time scale PTOA models, their relevance in injury models of short time scale is uncertain. For these reasons, we chose to focus on replicating experimental findings with a minimal number of cell crosstalk-related parameters, which are explored elsewhere [3,48,52–54].”

**REVIEWER #3**

**GENERAL COMMENT:** The authors developed a simplified mechanobiological model that combines mechanical loading effects with cellular responses in cartilage. The four elements of the model are cell "damage", proteolytic activity, fluid flow, and biosynthetic responses. The model predicted that fluid flow drives aggrecan loss near lesions, an effect outweighing that of proteolytic activity. The model also predicted biosynthetic responses outweigh degradation in deeper tissue.

Parts of the model involve speculation, mostly well acknowledged by the authors. The reviewer would have made different choices for several parts of the model. However, the reviewer is enthusiastic about this as a founding contribution in an important area and has only a few minor, optional suggestions for the packaging of the final manuscript. Would the authors kindly consider adding discussion about the following limitations and adding a few notes about why specific choices were made? In summary, this is a very interesting paper and the reviewer is highly enthusiastic.

**AUTHOR RESPONSE:** The Authors thank the Reviewer for the enthusiastic comments and constructing suggestions. We have updated the manuscript accordingly, outlined below.

**REVIEWER COMMENT (1):** The model only includes two cell populations ("healthy" and "damaged"), which misses mechanisms like apoptosis, necrosis, oxidative stress, or hypertrophy. How might more realistic cell models be incorporated?

**AUTHOR RESPONSE:** Thank you for the insightful question. More realistic cell models should incorporate different variables for each “damaged/non-healthy” cell population (such as, apoptotic, necrotic, hypertrophic, and cells experiencing oxidative stress) instead of combining them into only one variable. This approach has been adopted in previous cell–tissue-level models [16–19]. However, introduction of new cellular variables necessitates also estimation of proper rate terms for production/loss of different cell populations with links to known (or hypothesized) mechanism(s) regulating these processes. This could prove challenging given the lack of experimental data. A potential approach to define the rate terms for how different cell populations evolve in a given mechanoinflammatory environment is to combine intra-cellular signaling models [20] to the biomechanical stress–strain/diffusion–reaction models. Please see also our response to the comment (3) by Reviewer #2.

**AUTHOR ACTION:** Discussion has been updated.

*Discussion* – *(pages 15–16, lines 328–343):* “A limitation of this computational model is the inclusion of only two cell populations, “healthy” and “damaged”. Cartilage mechanoinflammation is far more complex, involving *e.g.* (…) oxidative stress, mitochondrial dysfunction, apoptosis/necrosis, and cell hypertrophy [12,18,30,32,74]. (…) Modeling-wise the cellular variables and associated production/loss rates for different cell populations (as in Eq. (4)) may be implemented as linked to a certain, hypothesized mechanism or as basal sink/source terms when the real mechanism is not fully known. This approach has been adopted in previous cell–tissue-level models investigating cell death, hypertrophy, and oxidative stress, with comparison to experiments as far as the available data allow [3,53,54,75]. The challenge of estimating how the cell population concentrations evolve in different mechanoinflammatory environments may also be explored with intra-cellular signaling models [76], the linking of which to reaction–diffusion models remains to be established.”

**REVIEWER COMMENT (2):** The model omits tissue responses to injury-related pro-inflammatory cytokines and their effects on mechanical properties, which are known to affect osteoarthritis progression. What challenges led the authors to ignore these?

**AUTHOR RESPONSE:** We fully acknowledge that inflammation is an integral part of the injury response. However, here we focused on establishing the first version of the mechanoinflammatory modeling framework with *chondrocyte-driven* responses, starting with *mechanical overloading*-related mechanisms (continuation from our previous work without explicitly modeled chondrocytes [21]). Inflammation has previously been incorporated with exogenous cytokine diffusion [6], and steps have been taken to combine mechanics- and cytokine-driven cell responses [17,18]. Implementing pro-inflammatory cytokines to the current framework is possible (ongoing work of ours), although rather theoretical hypotheses about the interplay of biomechanical and inflammatory mechanisms are needed in injury–cyclic loading–inflammation models (*e.g.*, aggrecanase release rates due to different mechanisms). These are challenging to justify and validate with scarce experimental data, leading us to ignore cytokines — for now. Further challenges to tackle include understanding how the healthy *vs.* different “damaged” cell populations respond to cytokines (release of ADAMTS, MMPs) and cyclic loading (aggrecan biosynthesis rates), possible advection of cytokines, and tissue softening (*e.g.*, collagen) especially when high concentrations of cytokines are present (please see also our response to comment (1) by Reviewer #1).

**AUTHOR ACTION:** Discussion has been updated.

*Discussion* – *(page 16, lines 347–353):* “The model also omits the tissue responses to injury-related release and diffusion/advection of pro-inflammatory cytokines [13,49]. While exogenous cytokines have been included in previous models [3,53], their involvement introduces new difficult-to-validate parameters regarding healthy *vs.* damaged cell responses to cytokines (ADAMTS release, aggrecan biosynthesis) and relative magnitude of mechanics- *vs.* cytokine-driven catabolism. Justification and experimental comparison of these cell response parameters were out of scope of the current study.”

*Discussion* – *(pages 17–18, lines 383–393):* “Degradation softens cartilage, potentially leading to locally increasing strains over time. (…) Rigorous implementation of collagen failure to the current time-dependent mechanoinflammatory model would necessitate use of biomechanical tests, collagen content maps, and estimates of matrix metalloproteinase activity at several time points which is a topic for future work. (…) While tissue softening may not be a significant factor over 12 days, it becomes important over longer times in joint-level models.”

**REVIEWER COMMENT (3):** The model presumes an initial injury. What additional challenges might be faced with adding injury initiation?

**AUTHOR RESPONSE:** Properly modeling the injury initiation (and following lesion propagation) is not trivial, as it requires not only information on the tissue behavior under high-rate loading but also a different numerical formulation. First, the inputs to the model should include carefully, non-destructively measured/estimated (localized) sample-specific material properties, especially collagen orientation and stiffness, and tissue geometry [15] (please see also our response to comment (11) by Reviewer #1). Second, the corresponding numerical formulation for the material behavior (including defining fracture energy in porous composite structures under high-rate loading) combined with damage initiation assumptions (*e.g.*, collagen failure criteria, see response to comment (1) by Reviewer #1) should be approached with extended or cohesive finite element modeling methods (instead of conventional FEM as in the current study). These advanced methods were out of scope of the current study, but lesion initiation and propagation are part of our ongoing work.

**AUTHOR ACTION:** Discussion has been updated.

*Discussion* – *(pages 16–17, lines 358–361):* “Information on sample-specific geometry and material properties under high-rate loading, as well as corresponding numerical formulations for material behavior and damage initiation (*e.g.*, collagen failure), are all necessary for properly modeling lesion formation/propagation for instance with extended or cohesive finite element modeling (omitted in this study) [78,79].”

**REVIEWER COMMENT (4):** Timescales are of course totally arbitrary when you don't have a great lock on rate constants. Why did the authors choose to simulate over only 12 days? This reviewer is no cow, but his own osteoarthritis has developed over years. How did the authors come up with these timescales?

**AUTHOR RESPONSE:** From non-cow to non-cow, thank you for the question! This *ex vivo* experimental setup (that served as the basis for the computational model) has been designed on purpose to replicate features of the earliest stages of post-traumatic osteoarthritis progression (cellular, compositional, gene expression changes similar to the real disease), but in an accelerated form to accommodate reasonable time of lab work (days instead of years per single experiment) and without killing all the cells. The 12-day timescale is long enough to observe changes in the aggrecan content with cells still living (though longer culture times are possible) [5,11,22]. Moreover, we had the necessary aggrecan content maps at different time points available only up until day 12, so we chose to simulate over 12 days. For longer time periods that would translate to the human disease *in vivo*, more experimental/clinical data is needed for model calibration (please also see our response to comment (2) by Reviewer #1).

**AUTHOR ACTION:** Discussion and Methods sections have been updated.

*Methods* – *(page 19, lines 407–409):* “The experiments have been described in detail in two previous studies investigating localized aggrecan loss, which is observable already after 12 days in this experimental model of early-stage PTOA [7,14]. This study utilizes a subset of those data.”

*Discussion* – *(page 17, lines 373–381):* “Therefore, new explant culture/bioreactor studies should be conducted, not only with immature cartilage but also with mature (human cadaver) cartilage, in order to fully calibrate the numerical representation of the underlying mechanoinflammation. Another approach is organ-on-a-chip models with human cells, providing added control over desired mechanobiological processes compared to *ex vivo* models [80,81]. To further promote the translational ability of the model to the knee joint-level, the simulated aggrecan depletion should be confirmed in joint-level mechanoinflammatory models (*e.g.*, comparison to quantitative MRI maps at baseline and several follow-up time points [59]) and compared against matrix biomarkers released to synovial fluid or systemic circulation [35,82].”

**References:**

1.	Elahi SA, Castro-Viñuelas R, Tanska P, Korhonen RK, Lories R, Famaey N, et al. Contribution of collagen degradation and proteoglycan depletion to cartilage degeneration in primary and secondary osteoarthritis: an in silico study. Osteoarthritis Cartilage. 2023;31: 741–752. doi:10.1016/j.joca.2023.01.004

2.	Mononen M, Tanska P, Isaksson H, Korhonen R. A novel method to simulate the progression of collagen degeneration of cartilage in the knee: Data from the osteoarthritis initiative. Sci Rep. 2016;6: 21415. doi:10.1038/srep21415

3.	Pratta MA, Yao W, Decicco C, Tortorella MD, Liu RQ, Copeland RA, et al. Aggrecan Protects Cartilage Collagen from Proteolytic Cleavage. Journal of Biological Chemistry. 2003;278: 45539–45545. doi:10.1074/jbc.M303737200

4.	Li Y, Wang Y, Chubinskaya S, Schoeberl B, Florine E, Kopesky P, et al. Effects of insulin-like growth factor-1 and dexamethasone on cytokine-challenged cartilage: Relevance to post-traumatic osteoarthritis. Osteoarthritis Cartilage. 2015;23: 266–274. doi:10.1016/j.joca.2014.11.006

5.	Eskelinen ASA, Florea C, Tanska P, Hung HK, Frank EH, Mikkonen S, et al. Cyclic loading regime considered beneficial does not protect injured and interleukin-1-inflamed cartilage from post-traumatic osteoarthritis. J Biomech. 2022;141: 111181. doi:10.1016/j.jbiomech.2022.111181

6.	Kar S, Smith D, Gardiner B, Li Y, Wang Y, Grodzinsky A. Modeling IL-1 induced degradation of articular cartilage. Arch Biochem Biophys. 2016;594: 37–53. doi:10.1016/j.abb.2016.02.008

7.	Henao-Murillo L, Pastrama MI, Ito K, van Donkelaar CC. The relationship between proteoglycan loss, overloading-induced collagen damage, and cyclic loading in articular cartilage. Cartilage. 2019; 1–12. doi:10.1177/1947603519885005

8.	Thibault M, Poole A, Buschmann M. Cyclic compression of cartilage/bone explants in vitro leads to physical weakening, mechanical breakdown of collagen and release of matrix fragments. Journal of Orthopaedic Research. 2002;20: 1265–1273. doi:10.1016/S0736-0266(02)00070-0

9.	Ayala S, Matan SO, Delco ML, Fortier LA, Cohen I, Bonassar LJ. Degradation of lubricating molecules in synovial fluid alters chondrocyte sensitivity to shear strain. Journal of Orthopaedic Research. 2024. doi:10.1002/jor.25960

10.	Kisiday JD, Jin M, DiMicco MA, Kurz B, Grodzinsky AJ. Effects of dynamic compressive loading on chondrocyte biosynthesis in self-assembling peptide scaffolds. J Biomech. 2004;37: 595–604. doi:10.1016/j.jbiomech.2003.10.005

11.	Szapary HJ, Flaman L, Frank E, Chubinskaya S, Dwivedi G, Grodzinsky AJ. Effects of dexamethasone and dynamic loading on cartilage of human osteochondral explants challenged with inflammatory cytokines. J Biomech. 2023;149. doi:10.1016/j.jbiomech.2023.111480

12.	Sah R, Kim Y, Doong J, Grodzinsky A, Plaas A, Sandy J. Biosynthetic response of cartilage explants to dynamic compression. Journal of Orthopaedic Research. 1989;7: 619–636. doi:10.1002/jor.1100070502

13.	Patwari P, Gaschen V, James I, Berger E, Blake S, Lark M, et al. Ultrastructural quantification of cell death after injurious compression of bovine calf articular cartilage. Osteoarthritis Cartilage. 2004;12: 245–252. doi:10.1016/j.joca.2003.11.004

14.	Li Y, Frank EH, Wang Y, Chubinskaya S, Huang HH, Grodzinsky AJ. Moderate dynamic compression inhibits pro-catabolic response of cartilage to mechanical injury, tumor necrosis factor-α and interleukin-6, but accentuates degradation above a strain threshold. Osteoarthritis Cartilage. 2013;21: 1933–1941. doi:10.1016/j.joca.2013.08.021

15.	Jönsson V, Orozco GA, Pierantoni M, Dejea H, Gustafsson A, Grassi L, et al. Influence of articular cartilage sample geometry on mechanical response and properties using finite element simulation. J Biomech. 2024;176. doi:10.1016/j.jbiomech.2024.112323

16.	Kapitanov G, Wang X, Ayati B, Brouillette M, Martin J. Linking cellular and mechanical processes in articular cartilage lesion formation: A mathematical model. Front Bioeng Biotechnol. 2016;4: 1–22. doi:10.3389/fbioe.2016.00080

17.	Rahman MM, Watton PN, Neu CP, Pierce DM. A chemo-mechano-biological modeling framework for cartilage evolving in health, disease, injury, and treatment. Comput Methods Programs Biomed. 2023;231. doi:10.1016/j.cmpb.2023.107419

18.	Kosonen JP, Eskelinen ASA, Orozco GA, Nieminen P, Anderson DD, Grodzinsky AJ, et al. Injury-related cell death and proteoglycan loss in articular cartilage: Numerical model combining necrosis, reactive oxygen species, and inflammatory cytokines. PLoS Comput Biol. 2023;19: e1010337. doi:10.1371/journal.pcbi.1010337

19.	Kosonen JP, Eskelinen ASA, Orozco GA, Coleman MC, Goetz JE, Anderson DD, et al. Mechanobiochemical finite element model to analyze impact-loading-induced cell damage, subsequent proteoglycan loss, and anti-oxidative treatment effects in articular cartilage. Biomech Model Mechanobiol. 2025. doi:10.1007/s10237-025-01961-8

20.	Lesage R, Ferrao Blanco MN, Narcisi R, Welting T, van Osch GJVM, Geris L. An integrated in silico-in vitro approach for identifying therapeutic targets against osteoarthritis. BMC Biol. 2022;20. doi:10.1186/s12915-022-01451-8

21.	Orozco G, Tanska P, Florea C, Grodzinsky A, Korhonen R. A novel mechanobiological model can predict how physiologically relevant dynamic loading causes proteoglycan loss in mechanically injured articular cartilage. Sci Rep. 2018;8: 15599. doi:10.1038/s41598-018-33759-3

22.	Dwivedi G, Flaman L, Alaybeyoglu B, Struglics A, Frank EH, Chubinskaya S, et al. Inflammatory cytokines and mechanical injury induce post‑traumatic osteoarthritis‑like changes in a human cartilage‑bone‑synovium microphysiological system. Arthritis Res Ther. 2022;24: 1–19. doi:10.1186/s13075-022-02881-z
